# A Cross-Sectional Comparative Evaluation of the Forced Oscillation Technique and Spirometry in Patients Suspected of Having Obstructive Airway Disease at a Tertiary Care Centre

**DOI:** 10.7759/cureus.110115

**Published:** 2026-06-02

**Authors:** Shubhra Srivastava, Sachin Kumar, Ved Prakash, Mohammad Arif, Anurag Tripathi, Deepak Sharma, Aparna Chinmai, Sandeep Kumar, Hemant Kumar, Mrityunjaya Singh

**Affiliations:** 1 Pulmonary and Critical Care Medicine, King George's Medical University, Lucknow, IND; 2 Respiratory Medicine, Dr. Ram Manohar Lohia Institute of Medical Sciences, Lucknow, IND

**Keywords:** forced oscillation technique, impulse oscillometry, obstructive airway disease, small airway disease, spirometry

## Abstract

Introduction

Spirometry, the standard diagnostic tool for obstructive airway disease (OAD), is limited by effort dependency and poor sensitivity for small airways dysfunction. The forced oscillation technique (FOT) provides a non-invasive, effort-independent physiological assessment. This study aimed to evaluate the baseline physiological correlation between FOT and spirometry indices in suspected OAD and assess the role of FOT in patients unable to perform spirometry.

Methods

This cross-sectional observational study enrolled 201 patients presenting with clinical suspicion of OAD at a tertiary care pulmonology department. All participants underwent FOT and spirometry with post-bronchodilator assessment. Airflow obstruction and bronchodilator responsiveness on spirometry were defined as per European Respiratory Society/American Thoracic Society (ERS/ATS) guidelines. FOT abnormalities and bronchodilator responsiveness were interpreted using previously published literature-based thresholds for oscillometric parameters. Body plethysmography was additionally performed. Patients were stratified into three groups: Group A, obstruction on both spirometry and FOT, n=135 (67.2%); Group B, normal spirometry with obstruction on FOT, n=57 (28.4%); and Group C, unable to perform spirometry with obstruction on FOT, n=9 (4.5%). Statistical analysis was performed using SPSS version 24.0.

Results

FOT peripheral indices, particularly AX, X5, and R5-R20, showed the strongest correlations with percentage of predicted forced expiratory volume in one second (FEV₁%). Bronchodilator responsiveness was highest in Group B. FOT parameters worsened progressively with increasing obstruction severity, and percentage changes in FOT indices after bronchodilator administration correlated strongly with FEV₁% change.

Conclusion

FOT demonstrated excellent feasibility and identified airway obstruction in patients with preserved spirometry, supporting its complementary role in the physiological assessment of OAD. Peripheral oscillometry indices serve as valuable markers of small airways dysfunction and bronchodilator responsiveness.

## Introduction

Mechanical properties of the lung are important determinants and indicators of lung function and therefore help in the diagnosis and monitoring of several respiratory disorders. The most common among these are obstructive airway diseases (OAD), which affect almost 10% of the world population, with an even higher estimated burden in India due to indoor and outdoor pollution [1-3]. Asthma and chronic obstructive pulmonary disease (COPD) together account for the majority of OAD cases [4].

Measurement of lung function is an integral component of decision-making in patients with OAD. It assists in establishing a specific diagnosis, grading disease severity to guide pharmacotherapy, determining prognosis, and monitoring response to treatment [5]. Pulmonary function testing, particularly spirometry, has long served as the cornerstone of such evaluation. Spirometry provides key indices such as forced expiratory volume in one second (FEV₁), forced vital capacity (FVC), and ratio of forced expiratory volume in one second to forced vital capacity (FEV₁/FVC) ratio, which play a crucial role in the diagnosis and management of asthma and COPD [6]. A restrictive pattern on spirometry prompts further testing with lung volumes and diffusing capacity to identify restrictive diseases such as interstitial lung disease [7].

However, spirometry has important limitations. It requires forceful expiratory and inspiratory maneuvers and thus adequate cooperation, which is often lacking in young children, the elderly, and those with physical or cognitive impairment [4]. Results are effort-dependent and technique-sensitive, and the method predominantly reflects flow limitation in larger airways, with limited sensitivity for small airways dysfunction [8].

The forced oscillation technique (FOT) has emerged as a promising and increasingly used non-invasive method for assessing respiratory mechanics without requiring active patient participation. Based on the application of external pressure oscillations, as first described by DuBois AB et al. in 1956 [9], it allows easy performance, measures resistance and reactance over a range of frequencies, and may reflect changes related to small-airway dysfunction [8, 10-12]. Previous studies comparing spirometry and FOT have reported variable correlations and differing sensitivity for detecting airway abnormalities, particularly in small-airway dysfunction. Despite the increasing use of FOT in OAD, important gaps remain regarding its comparative utility with spirometry, particularly for small-airway dysfunction and bronchodilator responsiveness (BDR). Data from Indian populations are limited, and the role of FOT in symptomatic patients with preserved spirometry or an inability to perform acceptable spirometry remains incompletely understood.

This study aimed to evaluate the baseline physiological correlation between the FOT and spirometry indices in suspected obstructive airway disease (primary objective). Secondary exploratory objectives included assessing FOT correlations with BDR, plethysmography residual volume/total lung capacity (RV/TLC) ratios, and serum CRP levels.

This article was previously presented as a meeting abstract at the 59th Annual Convention of the Indian College of Allergy, Asthma and Applied Immunology held in Lucknow, Uttar Pradesh, from October 30 to November 2, 2025.

## Materials and methods

Study design and setting

This was a cross-sectional observational study conducted over a period of 1.5 years in the Department of Pulmonary and Critical Care Medicine at a tertiary care teaching hospital. The study protocol was reviewed and approved by the Institutional Ethics Committee, and written informed consent was obtained from all participants prior to enrolment.

Study participants

The study population comprised patients aged 12 years and above presenting to the Pulmonary and Critical Care Medicine outpatient department with symptoms suggestive of OAD, including shortness of breath, chronic cough, wheezing, or dyspnoea, with or without a history of smoking, biomass exposure, or prior antitubercular therapy. Inclusion criteria were age 12 years or above, suspected obstructive lung disease presenting with shortness of breath, absence of active infection for at least four weeks prior to enrolment, and no prior bronchodilator therapy.

Patients were excluded if they were younger than 12 years; were unable to perform FOT correctly; had significant comorbidities such as renal or liver failure, severe congestive heart failure, severe cardiomyopathy, or a history of life-threatening arrhythmias; had concurrent oncological disorders or claustrophobia; were pregnant; had acute exacerbation of disease or clinically significant hypoxia requiring urgent management; had recent respiratory infection, active tuberculosis, interstitial lung disease, or any contraindication to spirometry; were already on bronchodilator therapy; or refused or withdrew consent.

The sample size was calculated to detect a statistically significant correlation between FOT parameters and spirometric indices using the Pearson correlation coefficient-based formula, where Z corresponds to a 95% confidence level (1.96), power of 80% (0.84), and r represents the expected correlation coefficient. An anticipated correlation coefficient of r = 0.20 was assumed based on previous studies demonstrating weak to moderate correlations between oscillometric parameters and spirometric indices in patients with OAD [11-14]. Substituting these values into the formula yielded a minimum required sample size of approximately 189 participants. To account for potential dropouts, incomplete data, and suboptimal test performance, the final sample size was increased to 201 participants.

Data collection procedure

Consecutive patients fulfilling the eligibility criteria were recruited during the study period. A standardized proforma was used to record demographic details and clinical history, including symptom profile, smoking history, occupational and environmental exposures, and relevant comorbidities. A focused physical examination, with emphasis on respiratory findings, was performed in all participants.

Pulmonary function testing was then carried out. Spirometry was performed using a calibrated device in accordance with ERS/ATS Technical Standards for Spirometry (2022) [15]. Parameters recorded included FEV₁, FVC, and the FEV₁/FVC ratio. Post-bronchodilator values were obtained 15 minutes after administration of 400 mcg salbutamol via a metered-dose inhaler with spacer. Spirometry was attempted in all patients, and those unable to generate acceptable and reproducible efforts were documented. Airflow obstruction was defined as a post-bronchodilator FEV₁/FVC ratio <70%. BDR was interpreted according to updated ERS/ATS recommendations, with a positive bronchodilator response defined as an increase in FEV₁ or FVC of >10% of the predicted value following bronchodilator administration.

All 201 patients also underwent FOT assessment using the tremoflo® C-100 Airwave Oscillometry System, a FOT-based device, in accordance with manufacturer recommendations and standard operating procedures. Daily calibration verification and quality checks were performed prior to testing. At least three technically acceptable recordings were obtained in a seated position with nose clips, ensuring stable tidal breathing through a mouthpiece. Recordings affected by coughing, swallowing, vocalization, air leak, or irregular breathing artifacts were excluded from analysis. Recorded parameters included R5 (resistance at 5 Hz), R20 (resistance at 20 Hz), R5-R20 (frequency-dependent resistance), X5 (reactance at 5 Hz), and AX (area of reactance). Obstruction on FOT was defined using threshold values derived from existing literature: R5 >4 cmH₂O/L/s, R20 >3 cmH₂O/L/s, R5-R20 >1 cmH₂O/L/s, X5 <-1 cmH₂O/L/s, and AX >4 cmH₂O/L [16]. BDR on FOT was interpreted according to cut-offs described by Van der Burg et al., with BDR considered positive when there was a reduction in R5 >20%, R20 >18%, R5-R20 >25-45%, and an improvement in X5 >25% and AX >40% [17]. Patients fulfilling these cut-off criteria for a given parameter were designated BDR-positive for that parameter, while the remainder were considered BDR-negative.

Body plethysmography was performed in patients using a body plethysmograph as per ATS/ERS recommendations [18]. The lung volumes measured included RV, TLC, RV/TLC ratio, and functional residual capacity (FRC). Plethysmographic data provided additional information on lung hyperinflation and air trapping, with RV/TLC >40% taken as the cut-off for air trapping. All pulmonary function tests were performed by trained technicians following standard operating procedures, with daily calibration verification and routine quality control measures maintained throughout the study period.

All 201 patients successfully performed FOT. Spirometry and body plethysmography were performed by 192 patients (95.5%), while nine patients (4.5%) were unable to perform these two tests. Abnormal FOT parameters suggestive of airway obstruction were observed in all 201 patients, whereas spirometry demonstrated airflow obstruction in only 135 out of 192 patients. Notably, 57 patients had normal spirometry results despite showing obstruction on FOT. Based on these findings, participants were categorized into three groups: Group A (135) included patients with obstruction confirmed by both spirometry and FOT; Group B (57) comprised those with normal spirometry but abnormal FOT parameters suggestive of airway obstruction; and Group C (9) included patients who could only perform FOT. Where clinically indicated, additional investigations such as CRP, chest X-ray, and/or CT thorax were also obtained.

All collected data were entered into a structured Excel-based master chart. Patient identifiers were anonymized using unique study codes to ensure confidentiality, and only authorized study personnel had access to identifiable information.

Statistical analysis

The data were analysed using SPSS version 24.0. Continuous variables were summarized as mean ± SD or as median with interquartile range, and categorical variables as frequencies and percentages. Column and bar charts were used for graphical representation. Normality of continuous variables was assessed using the Shapiro-Wilk test. Pairwise comparisons for continuous variables were performed using the independent-samples t-test for normally distributed data and the Mann-Whitney U test for non-normal data, where appropriate, while the Chi-square test or Fisher’s exact test was used for categorical variables when more than 20% of cells had expected counts less than five. Correlations between continuous variables were assessed using Pearson’s or Spearman’s correlation coefficients, as appropriate, and a p-value <0.05 was considered statistically significant.

## Results

Of all 201 study participants, the mean age was 41.69 ± 19.66 years, with an age range of 12 to 78 years. The study population was nearly equally distributed by gender, with 101 males (50.2%) and 100 females (49.8%). The mean BMI was 22.86 ± 4.69 kg/m², ranging from 13.40 to 38.30 kg/m². Assessment of risk factors showed that 33 patients (16.4%) had a history of antitubercular therapy (ATT) intake. Smoking was reported in 65 patients (32.3%), and exposure to biomass fuel was noted in 50 patients (24.9%). A significant proportion of patients (117, 58.2%) had a history of allergic symptoms.

Table 1 shows the socio-demographic and clinical distribution of patients across the three study groups defined by spirometry and FOT findings. Group A (n=135) had obstruction on both spirometry and FOT, Group B (n=57) had normal spirometry with obstruction on FOT, and Group C (n=9) was unable to perform spirometry but showed obstruction on FOT. The mean age was highest in Group C (53.44 years), followed by Group A (44.10 years) and Group B (35.23 years). Males predominated in Group B, 32 (56.14%), while females were more common in Groups A and C. BMI was similar in Groups A and B, approximately 23 kg/m², but lower in Group C (19.84 kg/m²). ATT intake and biomass fuel exposure were more frequent in Group A, smoking was most prevalent in Group C, and allergy history was highest in Group B, 46 (80.70%).

**Table 1:** Socio-demographic distribution and clinical features of study participants by study group. ATT: Antitubercular treatment.

Parameters		Group A (n=135)	Group B (n=57)	Group C (n=9)
Age	Mean ± SD	44.10 ± 19.39	35.23 ± 14.73	53.44 ± 30.66
	Min-Max	12-75	12-70	12-78
Gender	Male	65 (48.15%)	32 (56.14%)	3 (33.33%)
	Female	70 (51.85%)	25 (43.86%)	6 (66.67%)
BMI	Mean ± SD	23.08 ± 4.73	23.89 ± 4.45	19.84 ± 3.24
Risk factors	ATT intake	28 (20.74%)	3 (5.26%)	2 (22.22%)
	Smoking	50 (37.04%)	11 (19.30%)	6 (66.67%)
	Biomass fuel exposure	43 (31.85%)	7 (12.28%)	2 (22.22%)
	Allergy	72 (53.33%)	46 (80.70%)	3 (33.33%)
Clinical symptoms	Breathlessness	135 (100%)	57 (100%)	9 (100%)
	Cough	67 (49.63%)	41 (71.93%)	6 (66.67%)
	Wheeze	38 (28.15%)	10 (17.54%)	2 (22.22%)
	Expectoration	37 (27.41%)	15 (26.32%)	2 (22.22%)
	Rhinitis/sneezing	43 (31.85%)	44 (77.19%)	3 (33.33%)

The findings in Table 2 are presented primarily as descriptive physiological characteristics across study groups. Baseline spirometry and FOT parameters demonstrated clear physiological differences among the three groups. Group A showed reduced spirometric indices, with a mean FEV₁/FVC of 59.49 ± 10.01 and FEV₁% of 53.08 ± 13.66, reflecting OAD. In contrast, Group B had preserved spirometry (FEV₁/FVC 80.76 ± 5.85, FEV₁% 77.86 ± 12.30) despite respiratory symptoms, while Group C could not perform spirometry. FOT parameters were abnormal across all groups, with Group A showing higher resistance at 5 Hz (R5) (7.32 ± 2.69), higher area of reactance (AX) (47.86 ± 41.84), and more negative reactance at 5 Hz (X5) (-6.09 ± 2.33), reflecting peripheral airway obstruction. Notably, Group B and Group C also demonstrated elevated R5 and AX, suggesting airway dysfunction not detected by spirometry. RV/TLC was higher in Group A (41.71 ± 9.54), with air trapping present in 77 patients (57.04%), compared to Group B (36.20 ± 7.37), with air trapping in 20 patients (35.09%). BDR was highest in Group B, 45/57 (78.95%), followed by Group A, 66/135 (48.89%), and Group C, 3/9 (33.33%).

**Table 2:** Baseline spirometry and forced oscillation technique parameters across study groups. FEV₁: Forced expiratory volume in one second; FVC: Forced vital capacity; FEV₁/FVC: Ratio of forced expiratory volume in one second to forced vital capacity; FEV₁%: Percentage of predicted forced expiratory volume in one second; FVC%: Percentage of predicted forced vital capacity; BD: Bronchodilator; R5: Resistance at 5 Hz; R20: Resistance at 20 Hz; R5-R20: Frequency dependence of resistance; X5: Reactance at 5 Hz; Fres: Resonant frequency; AX: Area of reactance; RV: Residual volume; TLC: Total lung capacity; RV/TLC: Ratio of residual volume to total lung capacity, used to assess air trapping.

Parameters		Group A	Group B	Group C
Spirometry, Mean ± SD (Min-Max)				
FEV₁/FVC		59.49 ± 10.01 (30.5-78.9)	80.76 ± 5.85 (70.0-92.7)	NA
FEV₁%		53.08 ± 13.66 (20.0-77.0)	77.86 ± 12.30 (42.0-98.0)	NA
FVC%		72.60 ± 12.35 (33.0-105.0)	81.86 ± 13.54 (45.0-104.0)	NA
Forced oscillation technique, Mean ± SD (Min-Max)				
R5		7.32 ± 2.69 (3.3-17.5)	6.37 ± 2.40 (3.2-14.5)	7.58 ± 3.25 (5.0-14.9)
R20		5.00 ± 1.93 (1.6-12.2)	4.74 ± 1.95 (0.8-11.2)	5.20 ± 1.97 (3.7-9.9)
R5-R20		2.31 ± 1.68 (0.0-8.2)	1.63 ± 1.16 (0.2-6.8)	2.39 ± 2.44 (0.1-8.2)
AX		47.86 ± 41.84 (6.2-179.3)	24.76 ± 27.91 (5.0-127.1)	43.92 ± 26.90 (15.4-86.8)
X5		-6.09 ± 2.33 (-11.6 to -1.7)	-3.84 ± 1.52 (-8.7 to -1.9)	-5.30 ± 1.95 (-8.8 to -2.7)
Body plethysmography, Mean ± SD (Min-Max)				
RV/TLC		41.71 ± 9.54 (25.0-67.0)	36.20 ± 7.37 (22.0-53.0)	NA
≤40, absent air trapping		58	37	NA
>40, present air trapping		77	20	NA
Bronchodilator response				
Present		66 (48.89%)	45 (78.95%)	3 (33.33%)
Absent		69 (51.11%)	12 (21.05%)	6 (66.67%)

**Figure 1:**
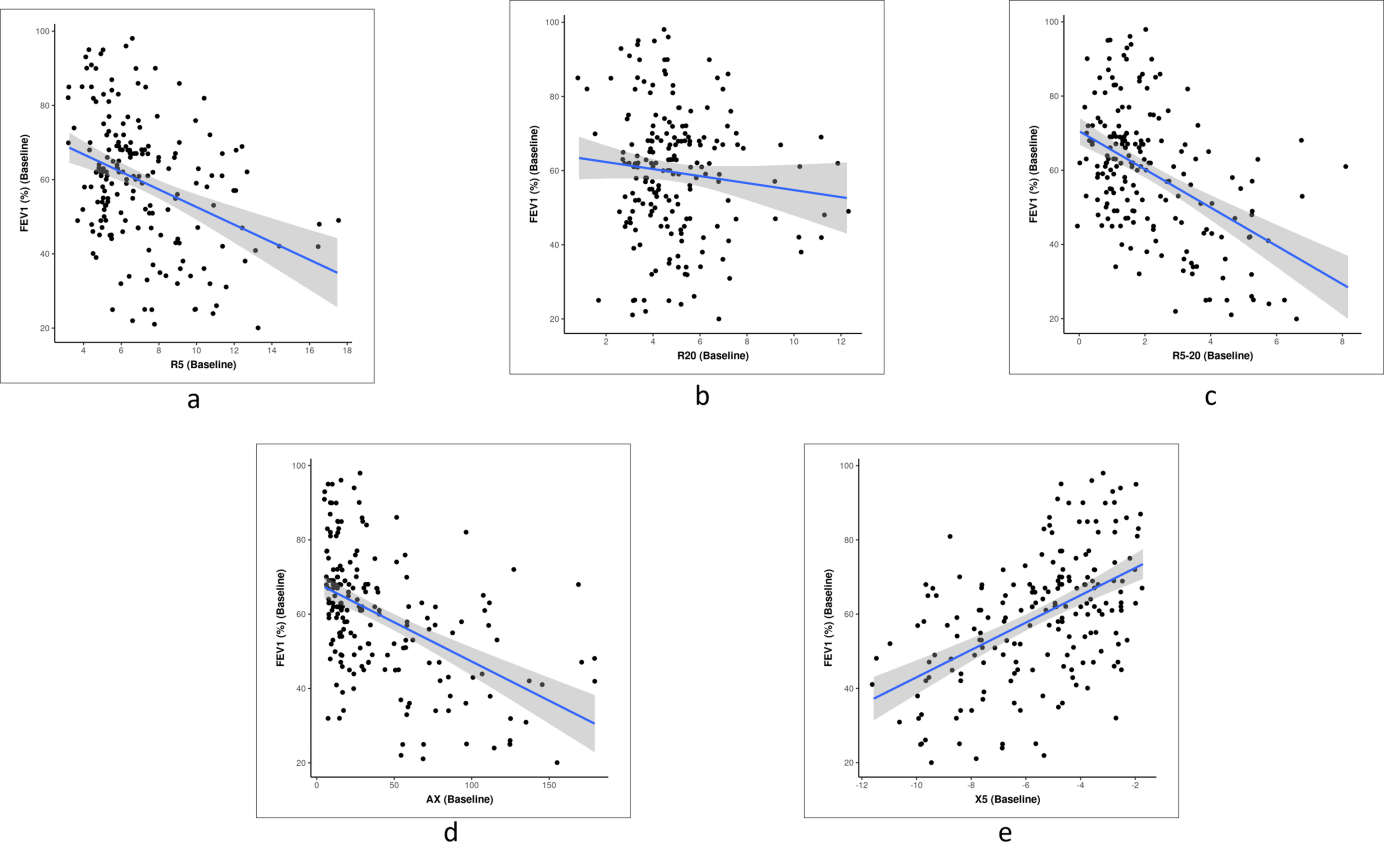
Scatter plots showing the correlation of FOT parameters with FEV₁%. Each dot represents an individual subject. The solid line represents the regression line, and the shaded area indicates the 95% CI. (a) A negative correlation is observed, with increasing R5 associated with lower FEV₁%; (b) a weak negative correlation is observed, with increasing R20 associated with lower FEV₁%; (c) a negative correlation is observed, with higher R5-R20 values associated with lower FEV₁%; (d) a negative correlation is observed, with increasing AX associated with lower FEV₁%; and (e) a positive correlation is observed, with less negative X5 values associated with higher FEV₁%. FOT: Forced oscillation technique; FEV₁: Forced expiratory volume in one second; FEV₁%: Percentage of predicted forced expiratory volume in one second; R5: Resistance at 5 Hz; R20: Resistance at 20 Hz; R5-R20: Difference between resistance at 5 Hz and resistance at 20 Hz / frequency dependence of resistance; AX: Area of reactance; X5: Reactance at 5 Hz; Hz: Hertz

Figure 1 illustrates the correlation between baseline FOT parameters and FEV₁%. R5 showed a moderate negative correlation with FEV₁% (rho = -0.34, p < 0.001), while R5-R20 demonstrated a moderate negative correlation (rho = -0.43, p < 0.001). AX exhibited the strongest negative correlation (rho = -0.51, p < 0.001), indicating high sensitivity to small airway dysfunction. X5 showed a moderate positive correlation with FEV₁% (rho = 0.49, p < 0.001), consistent with improved lung mechanics at higher FEV₁%. In contrast, R20 showed a weak and non-significant correlation (rho = -0.07, p = 0.355).

Following bronchodilator administration, patients in Groups A, B, and C were further classified into asthma and COPD subgroups. This classification was determined by integrating comprehensive clinical history, age of onset, smoking history, and clinical presentation, along with BDR, where a positive BDR supported an asthma phenotype in the appropriate clinical context, and a negative BDR supported a COPD phenotype. Correlation analysis revealed stronger associations between FOT parameters and airflow limitation in COPD than in asthma. In the asthma subgroup, only R5-R20 (rho = -0.25, p = 0.047) and AX (rho = -0.26, p = 0.034) showed weak but significant correlations with FEV₁%. In contrast, the COPD subgroup demonstrated moderate negative correlations for R5 (rho = -0.37, p = 0.001), R5-R20 (rho = -0.45, p < 0.001), and AX (rho = -0.47, p < 0.001), along with a significant positive correlation between X5 and FEV₁% (rho = 0.28, p = 0.013). As spirometric obstruction worsens, reflected by lower FEV₁%, peripheral lung reactance also declines and becomes more negative, directly reflecting an increase in small airway stiffness and capacitive load (Table 3).

**Table 3:** Correlation of baseline FEV₁% with FOT parameters in asthma and COPD subgroups among Group A patients. FOT: Forced oscillation technique; FEV₁: Forced expiratory volume in one second; R5: Resistance at 5 Hz; R20: Resistance at 20 Hz; R5-R20: Frequency dependence of resistance; AX: Area of reactance; X5: Reactance at 5 Hz; COPD: Chronic obstructive pulmonary disease; rho: Spearman’s correlation coefficient; p < 0.05: Statistically significant.

Correlation of baseline FOT parameters with FEV₁%	Asthma rho	Asthma p-value	COPD rho	COPD p-value
R5 vs FEV₁%	-0.11	0.363	-0.37	0.001
R20 vs FEV₁%	0.02	0.901	-0.14	0.241
R5-R20 vs FEV₁%	-0.25	0.047	-0.45	<0.001
AX vs FEV₁%	-0.26	0.034	-0.47	<0.001
X5 vs FEV₁%	0.03	0.795	0.28	0.013

FOT parameters worsened progressively with increasing severity of airflow obstruction. Mean R5 increased from 6.64 ± 2.02 in moderate obstruction to 8.98 ± 2.41 in very severe obstruction, while R5-R20 rose from 1.83 ± 1.49 to 4.91 ± 1.16. AX showed a marked increase from 33.63 ± 30.19 to 98.78 ± 35.12, and X5 became increasingly negative (-5.61 ± 2.09 to -7.96 ± 1.74). Significant correlations with FEV₁% were observed for R5 (r = -0.311), R5-R20 (r = -0.486), AX (r = -0.465), and X5 (r = 0.344), whereas R20 showed no significant association (r = -0.003) (Table 4).

**Table 4:** Characterization of airway obstruction severity using the correlation between FOT parameters and FEV₁%. FOT: Forced oscillation technique; FEV₁%: Percentage of predicted forced expiratory volume in one second; FOT: Forced oscillation technique; FEV₁: Forced expiratory volume in one second; FEV₁%: Percentage of predicted forced expiratory volume in one second; R5: Resistance at 5 Hz; R20: Resistance at 20 Hz; R5-R20: Frequency dependence of resistance; AX: Area of reactance; X5: Reactance at 5 Hz; Hz: Hertz; p < 0.05: Statistically significant.

FOT parameters	Moderate obstruction (FEV₁% 50-80%)	Severe obstruction (FEV₁% 30-50%)	Very severe obstruction (FEV₁% <30%)	Correlation coefficient	Statistical significance (p < 0.05)
Baseline R5	6.64 ± 2.02	8.36 ± 3.34	8.98 ± 2.41	-0.311	Yes
Baseline R20	4.82 ± 1.55	5.58 ± 2.32	4.07 ± 1.48	-0.003	No
Baseline R5-R20	1.83 ± 1.49	2.78 ± 1.54	4.91 ± 1.16	-0.486	Yes
Baseline AX	33.63 ± 30.19	64.40 ± 49.61	98.78 ± 35.12	-0.465	Yes
Baseline X5	-5.61 ± 2.09	-6.77 ± 2.58	-7.96 ± 1.74	0.344	Yes

Percentage changes in FOT parameters following bronchodilator administration showed strong concordance with FEV₁% improvement. The strongest correlation was observed for R5% change (rho = 0.75, 95% CI: 0.68-0.79), followed by X5% change (rho = 0.74), R5-R20% change (rho = 0.73), AX% change (rho = 0.72), and R20% change (rho = 0.63). All correlations were statistically significant (p < 0.001), supporting the utility of FOT in assessing bronchodilator response (Table 5).

**Table 5:** Correlation between percentage changes in FOT parameters and FEV₁ following bronchodilator administration in Group A. FOT: Forced oscillation technique; FEV₁: Forced expiratory volume in one second; FEV₁%: Percentage of predicted forced expiratory volume in one second; R5: Resistance at 5 Hz; R20: Resistance at 20 Hz; R5-R20: Frequency dependence of resistance; AX: Area of reactance; X5: Reactance at 5 Hz; Hz: Hertz; rho: Spearman’s correlation coefficient; p < 0.05, statistically significant.

Correlation of percentage change in FOT parameters with FEV₁% change	Spearman’s correlation coefficient (rho)	p-value
R5% change vs FEV₁% change	0.75 (95% CI: 0.68 to 0.79)	<0.001
R20% change vs FEV₁% change	0.63 (95% CI: 0.52 to 0.71)	<0.001
R5-R20% change vs FEV₁% change	0.73 (95% CI: 0.67 to 0.78)	<0.001
AX% change vs FEV₁% change	0.72 (95% CI: 0.65 to 0.77)	<0.001
X5% change vs FEV₁% change	0.74 (95% CI: 0.67 to 0.79)	<0.001

Baseline RV/TLC showed significant associations with FOT parameters, particularly those reflecting peripheral airway dysfunction. In the combined Group A+B, RV/TLC correlated positively with R5 (rho = 0.25), R5-R20 (rho = 0.55), and AX (rho = 0.39), and negatively with X5 (rho = -0.44) (all p < 0.001). Similar significant correlations were observed in Group A alone, whereas in Group B, only RV/TLC versus R5-R20 showed a significant association (rho = 0.34, p = 0.015), indicating a stronger linkage between air trapping and peripheral airway abnormalities in established obstruction (Table 6).

**Table 6:** Correlation between baseline RV/TLC and FOT parameters in Groups A and B combined, Group A, and Group B. RV/TLC: Residual volume/total lung capacity ratio; FOT: Forced oscillation technique; RV: Residual volume; TLC: Total lung capacity; R5: Resistance at 5 Hz; R20: Resistance at 20 Hz; R5-R20: Difference between resistance at 5 Hz and resistance at 20 Hz / frequency dependence of resistance; AX: Area of reactance; X5: Reactance at 5 Hz; rho: Spearman’s correlation coefficient; p-value: Probability value; Hz: Hertz.

Correlation	Combined Groups A and B rho	Combined Groups A and B p-value	Group A rho	Group A p-value	Group B rho	Group B p-value
RV/TLC vs R5	0.25	<0.001	0.21	0.012	0.27	0.062
RV/TLC vs R20	-0.11	0.132	-0.19	0.024	0.11	0.427
RV/TLC vs R5-R20	0.55	<0.001	0.58	<0.001	0.34	0.015
RV/TLC vs AX	0.39	<0.001	0.39	<0.001	0.18	0.204
RV/TLC vs X5	-0.44	<0.001	-0.39	<0.001	-0.12	0.414

**Figure 2:**
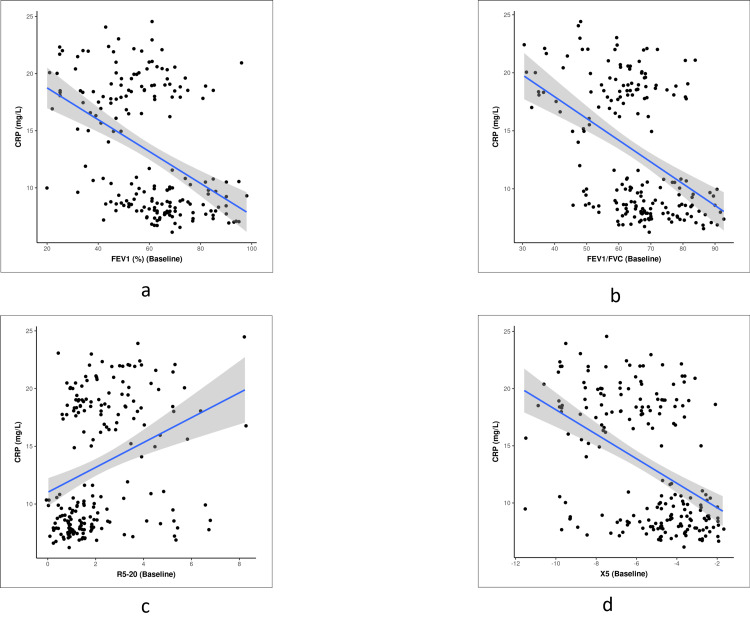
Correlation of baseline spirometry and FOT parameters with CRP (mg/L). Each dot represents an individual subject. The solid line represents the regression line, and the shaded area indicates the 95% CI. (a) Scatter plot showing a negative correlation between serum CRP and FEV₁ (% predicted), indicating that higher systemic inflammation is associated with greater airflow limitation; (b) scatter plot demonstrating a negative correlation between CRP and the FEV₁/FVC ratio, suggesting that increasing inflammation is linked with worsening airflow obstruction; (c) scatter plot showing a positive correlation between CRP and R5-R20, reflecting increased small airway resistance with higher CRP levels; and (d) scatter plot demonstrating a negative correlation between CRP and X5, indicating worsening peripheral airway reactance with increasing systemic inflammation. FOT: Forced oscillation technique; mg/L: Milligrams per liter; FEV₁: Forced expiratory volume in one second; FVC: Forced vital capacity; FEV₁/FVC: Ratio of forced expiratory volume in one second to forced vital capacity; R5: Resistance at 5 Hz; R20: Resistance at 20 Hz; R5-R20: Difference between resistance at 5 Hz and resistance at 20 Hz / frequency dependence of resistance; X5: Reactance at 5 Hz; Hz: Hertz.

Figure 2 demonstrates the relationship between pulmonary function and systemic inflammation. Among spirometry indices, FEV₁ (% predicted) showed a moderate negative correlation with CRP (rho = -0.43, 95% CI: -0.53 to -0.32, p < 0.001), with similar significant negative correlations for FVC (%) (rho = -0.39, p < 0.001) and the FEV₁/FVC ratio (rho = -0.42, p < 0.001). For FOT parameters, X5 correlated negatively with CRP (rho = -0.43, 95% CI: -0.54 to -0.31, p < 0.001), while R5-R20 (rho = 0.38, p < 0.001) and AX (rho = 0.29, p < 0.001) showed positive correlations; R20 showed a weak negative correlation (rho = -0.19, p = 0.007), whereas R5 had no significant association (rho = 0.10, p = 0.147), indicating that both spirometry and selected FOT parameters reflect systemic inflammatory burden.

## Discussion

The primary finding of our study highlights the high real-world feasibility of the FOT, which was successfully completed by every symptomatic patient in our cohort. Crucially, a subset of our population could only complete FOT, failing to perform conventional spirometry, which remains the clinical gold standard for diagnosing airflow obstruction. While FOT does not replace spirometry, this feasibility advantage is particularly vital given our cohort’s substantial risk-factor burden, including high rates of smoking, biomass exposure, and allergic symptoms. In these high-risk individuals, small-airway dysfunction often precedes overt spirometric changes. Methodologically, our inclusion of mixed obstructive phenotypes and a broad age range (12-78 years) contrasts with previous narrower studies, such as Heijkenskjöld RC et al. [19], demonstrating that our findings are applicable to heterogeneous, real-world clinical settings.

The clinical implications of the diagnostic discordance between standard spirometry and FOT are profound. In our cohort, nearly 30% of the symptomatic individuals who presented with completely normal, preserved spirometry values were found to have clear, objective airway obstruction when evaluated using FOT (Group B). This substantial rate of “oscillometry-only” abnormality strongly echoes the findings of Athavale T and Athavale A [13] in persistent asthma, confirming that oscillometry acts as a sensitive diagnostic safety net, exposing physiologically meaningful small-airway impairment that standard effort-dependent tests might miss. Clinically, this subgroup represents an early or predominantly small-airway phenotype. Despite having preserved forced expiratory volumes, their peripheral oscillometry indices were markedly altered, and they demonstrated a remarkably high rate of bronchodilator responsiveness. Rather than suggesting FOT as a replacement for conventional diagnostics, these findings demonstrate its crucial complementary role; integrating oscillometry alongside gold-standard spirometry allows clinicians to capture early, reversible peripheral airway dysfunction that would otherwise remain hidden.

Physiologically, our baseline correlation patterns reinforce existing literature regarding the superior sensitivity of peripheral reactance and resistance measures over central metrics. Throughout the entire dataset, spirometric airflow limitation aligned most robustly with total reactance area (AX) and distal reactance (X5), followed closely by frequency dependence of resistance (R5-R20) and total airway resistance (R5), while proximal resistance (R20) showed no meaningful association. This matches the structural patterns observed by Mou T et al. [14], where small-airway oscillometry indices correlated tightly with spirometric small-airway flows, with AX emerging as the most sensitive marker.

Our subgroup analysis further clarifies disease-specific behavior. While asthma showed only mild correlations with oscillometric indices, COPD demonstrated a far more pronounced, moderate coupling with airflow limitation across R5, R5-R20, and AX. This discrepancy suggests that oscillometric parameters may behave differently based on the underlying pathology, reflecting fixed structural remodeling and loss of lung elastic recoil in COPD versus the more variable, smooth-muscle-driven nature of asthma.

When examining severity stratification, we observed a progressive, stepwise worsening of peripheral airway resistance and reactance as spirometric airflow obstruction advanced. As obstruction shifted from moderate to very severe, R5, R5-R20, and AX escalated dramatically, while X5 became deeper and more negative. Conversely, central airway resistance (R20) remained flat and uncoupled from disease severity. These patterns indicate that distal, rather than proximal, indices are the true barometers of functional decline. Similar discriminative patterns for R5 have been noted in progressive adult asthma studies [20], validating its utility in tracking disease progression.

Finally, integrating advanced physiological markers revealed that established obstruction (Group A) is tied to significantly greater air trapping and hyperinflation than the early-stage Group B phenotype. The degree of air trapping correlated moderately with peripheral oscillometry indices, most notably with R5-R20, followed by X5 and AX, confirming that distal resistance changes are closely linked to mechanical gas trapping. Reassuringly, bronchodilator responsiveness showed strong physiological concordance between modalities, with the percentage improvement in expiratory volume tracking closely alongside improvements in total resistance, distal reactance, and frequency dependence.

Furthermore, our exploratory analysis revealed that systemic inflammatory burden, measured via CRP, was negatively associated with expiratory flow and distal reactance, while being positively associated with peripheral resistance metrics. This suggests that non-invasive oscillometry parameters may mirror not only mechanical small-airway dysfunction but also the underlying systemic inflammatory state. However, given the non-specific nature of CRP, these inflammatory links must be interpreted with caution.

This study has several strengths, including a robust sample size, near-equal sex distribution, and a wide age range, which collectively enhance its external validity. Simultaneous assessment using spirometry, body plethysmography, and FOT within the same symptomatic cohort allowed for a rare, direct head-to-head comparison of physiological abnormalities across distinct modalities. We successfully captured FOT data in all participants, including those entirely unable to tolerate the rigorous effort required for gold-standard spirometry, supporting FOT’s role as an objective, complementary tool in pulmonary function assessment.

However, several limitations must be acknowledged. Most notably, the lack of a healthy control group prevents the establishment of population-specific baseline reference values, while the single-centre, cross-sectional design precludes long-term causal inferences. Furthermore, the “oscillometry-only” abnormalities observed in Group B carry a risk of overinterpretation; prospective longitudinal data are required to confirm whether this phenotype reflects progressive small-airway disease or transient functional variations. Additionally, the small sample size of Group C limits subgroup precision, and hospital-based recruitment may introduce selection bias. Confounding variables, such as baseline medications, comorbidities, and exposure intensities, were not fully adjusted. Finally, because standardized diagnostic thresholds for FOT remain an evolving area, our reliance on fixed, literature-derived reference criteria may limit direct comparability with future multicentre trials.

## Conclusions

Our study demonstrates significant physiological discordance between FOT and spirometry in patients with suspected OAD. FOT identified peripheral airway abnormalities and high bronchodilator responsiveness in a notable subset of symptomatic patients with normal spirometric volumes (Group B). Rather than suggesting diagnostic failure of spirometry, these findings support the complementary, effort-independent role of FOT in enhancing the physiological assessment of OAD, particularly in patients with symptoms despite preserved spirometric measurements. Peripheral oscillometry indices were the most informative markers of airflow limitation and showed a clear relationship with increasing severity of obstruction, whereas proximal resistance measures contributed little to discrimination. Oscillometry also demonstrated strong correlations with bronchodilator responsiveness and meaningful associations with air trapping and selected inflammatory markers, supporting its complementary role alongside spirometry in the physiological assessment of OAD such as asthma and COPD.

Future studies should establish population-specific reference values for key FOT parameters, including AX, X5, and R5-R20, to define small-airway dysfunction and bronchodilator responsiveness across different phenotypes. Prospective, multicentre longitudinal research should evaluate whether oscillometry-only obstruction predicts outcomes such as symptoms, exacerbations, progression, and lung-function decline, and whether combined assessment using spirometry, FOT, lung volumes, and inflammatory biomarkers improves clinical decision-making and cost-effectiveness.
